# The Performance of a Customized Generative Pre-trained Transformer on the American Society for Surgery of the Hand Self-Assessment Examination

**DOI:** 10.7759/cureus.70205

**Published:** 2024-09-25

**Authors:** Jason C Flynn, Jacob Zeitlin, Sebastian D Arango, Nathaniel Pineda, Andrew J Miller, Tristan B Weir

**Affiliations:** 1 Department of Orthopaedic Surgery, Philadelphia Hand to Shoulder Center, Philadelphia, USA; 2 Department of Orthopaedics, Philadelphia Hand to Shoulder Center, Philadelphia, USA; 3 Department of Orthopaedic Surgery, Drexel University College of Medicine, Philadelphia, USA

**Keywords:** artificial intelligence, chatgpt, hand, natural language processing, orthopaedic

## Abstract

Introduction: Multimodal large language models (MLLMs), such as OpenAI’s ChatGPT (San Francisco, CA), have the potential to improve medical care delivery and education, although important shortcomings in accuracy and image interpretation have been noted. The aim of this study was to assess the multimodal performance of a ChatGPT model customized with hand surgery-specific knowledge.

Methods: A customized generative pre-trained transformer (GPT) was trained using peer-reviewed literature recommended by the American Society for Surgery of the Hand (ASSH). Questions were taken from the ASSH 2022 Self-Assessment Examination (SAE). GPT-4 and the customized GPT were asked text-based multiple-choice questions. The customized GPT was also asked image-containing questions, both with and without access to the image(s) associated with each question.

Results: A total of 192 questions were included. The customized GPT responded to the 119 text-only questions with greater accuracy than GPT-4 (107 (89.9%) versus 91 (76.5%), P = 0.001). Human examinees answered 87.3% (IQR: 71.6-93.7%) of the same text-based questions correctly. Of the 73 questions with images, the customized GPT answered 55 (75.3%) questions correctly, which dropped to 51 (69.9%) when the images were withheld (P = 0.317). The human examinees answered 87.2% (IQR: 79.4-95.4%) of image-based questions correctly.

Conclusion: Our findings suggest significant improvements in ChatGPT’s ability to answer text-based hand surgery questions with hand-specific training. ChatGPT is still limited in its ability to interpret images to answer questions related to hand conditions. These data show hand surgeons can create customized GPT models to provide tailored answers to specific questions, which may serve as the foundation for educational and clinical tools.

## Introduction

Artificial intelligence (AI) is a rapidly developing technology that has the potential to improve the accuracy of clinical decisions, streamline interactions with patients, and amplify the capabilities of providers [1-4]. Within medicine, and specifically in hand surgery, there are numerous applications to leverage AI in diagnosis, treatment, education, and surgical training [5-9]. ChatGPT (OpenAI, San Francisco, CA), a multimodal large language model (MLLM), utilizes human-like dialogue to allow users to quickly search through, interpret, and apply large volumes of internet data [10-15].

Prior research has evaluated ChatGPT’s performance on standardized examinations in general medicine, plastic surgery, and orthopedics [10-15]. In hand surgery, studies have shown inconsistent performance of ChatGPT on the American Society for Surgery of the Hand (ASSH) Self-Assessment Examination (SAE), answering 36-69% of questions correctly [15,16]. The performance of ChatGPT is especially poor (29%) when answering questions with associated images, but prior versions of ChatGPT did not have the ability to upload images for interpretation [16]. While prior studies have shown ChatGPT can provide comprehensive answers on a variety of hand surgery topics, authors have cautioned against the use of this technology in the clinical setting due to inconsistent and inaccurate responses [4,9,17]. When asked for references to support the responses by ChatGPT, it has been shown to even fabricate its sources [6,18,19]. Recent updates have enabled users to create customized ChatGPT models by providing specific resources and references to guide responses to questions. No studies have determined if a customized ChatGPT model trained with hand surgery references can improve the performance of ChatGPT when answering standardized hand surgery questions.

Therefore, the primary purpose of this study was to evaluate the performance of a customized generative pre-trained transformer (GPT) trained with hand surgery resources on the 2022 ASSH SAE compared with ChatGPT-4.0 (GPT-4) and human examinees. To evaluate the new image processing feature of ChatGPT, we sought to determine if the custom ChatGPT model had improved accuracy when provided with clinical and radiographic images on image-based questions. Finally, we sought to determine if the question difficulty or question topic was associated with the performance of ChatGPT.

## Materials and methods

ASSH SAE

The ASSH SAE (available online at: www.assh.org/s/self-assessment-examination) is a 200-question multiple-choice examination created by the SAE Committee of the ASSH each year [20]. The examination can be taken to receive Continuing Medical Education (CME) and/or Maintenance of Certification (MOC) credit. To receive CME credit, orthopedic and plastic surgery examinees must answer at least 50% correctly, while general surgery examinees must answer at least 75% correctly [20]. The 2022 ASSH SAE answer key provides the correct answer, an explanation, and references for each question. The 2022 ASSH SAE questions were screened to exclude video-based questions by a single reviewer. The remaining text and image-based questions were further stratified, resulting in 119 text-based questions and 73 image-based questions. Each question was transcribed from the official 2022 SAE and formatted as a stand-alone multiple-choice question to match the appearance on the official examination. For image-based questions, the associated image was retained during transcription.

Customized GPT training

OpenAI’s GPT builder feature allows users to customize GPT-4, augmenting its fund of knowledge via text-based prompts and file uploads. Using this feature, we included portable document formats (PDFs) for two to six relevant references per question provided by the ASSH SAE answer key. The customized GPT (GPT-Hand) was also provided the entirety of Green’s Operative Hand Surgery, 8th edition [21], textbook in PDF format. Prior to asking the multiple-choice question, GPT-Hand was instructed to reference the uploaded training knowledge and its innate internet training to synthesize answers to questions on the topic of hand surgery. The exact wording of these instructions remained identical throughout all parts of this analysis. These instructions were:

This GPT is an expert in the field of hand surgery. It utilizes its preexisting knowledge, paired with the uploaded documents, to answer complex questions regarding hand pathologies and surgery. When asked a question, this GPT always selects the answer which it believes to be best and provides a succinct explanation as to why.

Evaluating performance

To accomplish our primary objective, we compared the performance of GPT-Hand to GPT-4 on the 2022 ASSH SAE. The 119 text-based questions were each asked in separate chat boxes to ensure information from previous questions would not interfere with its response to the question being asked. The answer provided was recorded as correct or incorrect using the ASSH SAE answer key as a reference. The GPT was then prompted to provide the references it used to answer the question, and we recorded whether the reference was the same as that provided by the ASSH SAE answer key.

To determine if the image processing feature could help answer image-based questions, GPT-Hand was provided with question prompts with and without the clinical or radiographic images associated with each question (n = 73). Correct responses with and without the provided images were compared. GPT-Hand was also asked to provide the reference to support the answers to each question, and we recorded whether the reference was the same as that provided by the ASSH SAE answer key.

Finally, we sought to determine if question difficulty or question topic influenced ChatGPT’s ability to provide correct responses. Question difficulty was assessed by two raters using the ranking methodology described by Buckwalter et al. [22]. In the case of disagreement between the two raters, a third tie-breaker rater was used. Level 1 questions involved simple recall of discrete, factual knowledge; level 2 questions required interpretation and comprehension; and level 3 questions were the most difficult, involving the integration of knowledge to reach the correct answer [22]. Seven question categories were provided by the ASSH: basic science, bone and joint, neuromuscular, skin, vascular, ancillary, and miscellaneous [20]. In addition, ASSH provided discrimination index scores ranging from 0 to 1 to indicate the capacity of each question to differentiate high versus low performers on the actual examination.

Statistical analysis

The counts of each category and taxonomy with percentages were calculated, and proportions were compared using chi-squared tests. Using the human examinee data provided by the ASSH, the discrimination indices and percentages of correct responses by human examinees were compared between question types via Mann-Whitney U tests. The decision to separate text-only from image-containing questions was due to the novelty of image processing as a capability in GPT-4 [23]. Additionally, inter-rater reliability for question taxonomy (three possible levels) was determined by calculating the intraclass correlation coefficient (ICC) using a two-way mixed effects model. ICC values over 0.90 represent excellent reliability [24].

For text-only questions, the overall percentage of correct answers was compared between GPT-4 and GPT-Hand using McNemar’s test to consider matched pairs, and the performance of the two models according to question category and taxonomy levels were compared using Cochran’s Q test. For image-containing questions, performance of GPT-Hand was assessed under its normal conditions and compared to its performance when the question images were not included with the question prompt (text). This was done to assess the added benefit of image processing in the model.

A multivariable logistic regression analysis was performed to determine the factors associated with correct answers. A priori variables included the GPT model, question type (image-based versus text-only), question topic, question taxonomy level (i.e., difficulty), human examinee correct response rate, and question discrimination index. For all statistical tests, P < 0.05 was considered statistically significant.

## Results

Of the 192 questions included in the study, 119 were text-only and 73 were image-based questions (Table 1). The most common question taxonomy was level 1 (“recall”), comprising 56% of text-only and 37% of image-based questions. The “Bone and Joint” category was the most common image-based question category, while the “Other” category was most common for text-only questions.

**Table 1:** Attributes of text-only and image-based questions on the ASSH SAE. ASSH = American Society for Surgery of the Hand; SAE = Self-Assessment Examination. Categorical data are represented as question counts with percentages in parentheses, and continuous data are represented as medians with interquartile ranges in parentheses. Differences between text-only and image-based questions were assessed using chi-square tests. Statistical significance was defined as P < 0.05. * Soft tissue questions include neuromuscular, skin, and vascular categories.

Variable	Question type		P
	Text-only	Image-based	
Number of questions	119	73	—
Question category			
Basic science	24 (20.2)	10 (13.7)	0.002
Bone and joint	18 (15.1)	29 (39.7)	
Soft tissue*	25 (21.0)	12 (16.4)	
Other	52 (43.7)	22 (30.1)	
Question taxonomy			
Level 1 (recall)	66 (55.5)	27 (37.0)	0.019
Level 2 (interpretation)	23 (19.3)	26 (35.6)	
Level 3 (application)	30 (25.2)	20 (27.4)	
Human correct response rate (%)	87.3 (71.6 – 93.7)	87.2 (79.4 – 95.4)	0.340
ASSH discrimination index	0.16 (0.09 – 0.27)	0.15 (0.06 – 0.27)	0.330

For all text-only and image-based questions included in the study, GPT-Hand answered 162 (84.4%) questions correctly, and human examinees answered 87.3% (interquartile range (IQR) = 73.2-94.1%) correctly (Table 2). Regarding our primary objective, GPT-Hand provided significantly more correct answers than GPT-4 on text-only questions (89.9% versus 76.5%, respectively; P = 0.001). There were no significant differences in performance between the two models based on question category (P = 0.317) or taxonomy level (P = 0.317). The ICC for taxonomy level was 0.96 (95% CI: 0.95 - 0.97) for all questions, indicating excellent reliability.

**Table 2:** Comparison of performance between GPT-4 and GPT-Hand on the ASSH SAE. GPT = generative pre-trained transformer; ASSH = American Society for Surgery of the Hand; SAE = Self-Assessment Examination. Data are represented as medians with interquartile ranges in parentheses (for human correct response percentages), or as the number of questions answered correctly with the percentage in parentheses (for GPT models). Examinee data are based on the performance of hand surgeons (n = 1,864). Differences in performance between GPT models were assessed using McNemar’s test or Cochran’s Q test. Statistical significance was defined as P < 0.05. * Soft tissue questions include neuromuscular, skin, and vascular categories.

Variable	Number of questions	Respondent			P
		Human examinees	GPT-4	GPT-Hand	
All questions	192	87.3 (73.2 ­– 94.1)	—	162 (84.4)	—
Questions with images	73	87.2 (79.4 – 95.4)	—	55 (75.3)	—
All text-only questions	119	87.3 (71.6 – 93.7)	91 (76.5)	107 (89.9)	0.001
Category (text-only)					
Basic science	24	86.2 (67.7 – 93.4)	19 (79.2)	23 (95.8)	0.317
Bone and joint	18	89.3 (74.7 – 92.9)	14 (77.8)	16 (88.9)	
Soft tissue*	52	87.7 (72.2 – 93.9)	35 (67.3)	47 (90.4)	
Other	25	85.2 (70.6 – 94.1)	23 (92.0)	21 (84.0)	
Taxonomy (text-only)					
Level 1 (recall)	66	85.9 (70.6 – 93.2)	52 (78.8)	60 (90.9)	0.317
Level 2 (interpretation)	23	82.1 (67.8 – 95.0)	18 (78.3)	20 (87.0)	
Level 3 (application)	30	90.3 (83.2 – 93.9)	21 (70.0)	27 (90.0)	

When provided with the clinical or radiographic images associated with image-based questions, GPT-Hand answered 55 (75.3%) questions correctly, which dropped to 51 (69.9%) questions when the images were withheld (P = 0.317). The human examinees answered 87.2% (IQR, 79.4% - 95.4%) of the image-based questions correctly.

In the multivariable logistic regression for the predictors of correct responses, there was a significant negative association between the likelihood of a correct response from an MLLM and the discrimination index of a given question (OR = 0.95 (95% CI: 0.92 - 0.98) per 0.01 increase, P = 0.001) (Table 3). Questions that were text-only were also answered at higher accuracies by the MLLMs than questions containing images (OR = 3.73 (95% CI: 1.58 - 8.81), P = 0.003). However, the mean percentage of human examinees answering a question correctly was not a significant predictor of an MLLM answering the same question correctly (P = 0.370).

**Table 3:** Multivariable logistic regression for the predictors of correct responses on the ASSH SAE. GPT = generative pre-trained transformer; ASSH = American Society for Surgery of the Hand; SAE = Self-Assessment Examination; OR = odds ratio; CI = confidence interval. Odds ratios represent the expected probability of a correct response associated with each possible value of a variable, relative to a reference value. Each reference value, by definition, has an odds ratio of 1, signifying no change in probability. Statistical significance, determined using Wald tests, was defined as P < 0.05. * Soft tissue questions include neuromuscular, skin, and vascular categories.

Variable	OR (95% CI)	P
Model		
GPT-4	—	—
GPT-Hand	3.04 (1.41 – 6.56)	0.005
Type		
Image-containing	—	—
Text-only	3.73 (1.58 – 8.81)	0.003
Category		
Basic science	—	—
Bone and joint	0.95 (0.38 – 2.40)	0.910
Soft tissue*	0.55 (0.24 – 1.26)	0.160
Other	1.20 (0.45 – 3.19)	0.715
Taxonomy		
Level 1 (recall)	—	—
Level 2 (interpretation)	0.84 (0.43 ­– 1.64)	0.601
Level 3 (application)	0.71 (0.36 – 1.38)	0.307
ASSH discrimination index, per 0.01 points	0.95 (0.92 – 0.98)	0.001

## Discussion

The primary objective of this study was to examine the performance of a customized GPT in the context of both human trainees and GPT-4. GPT-Hand performed better than GPT-4 on text-only questions, indicating significant improvement was achieved by training ChatGPT on hand-specific resources. The performance of both GPT-Hand and GPT-4 fell within the interquartile range of human examinee performance on text-based questions (IQR = 71.6-93.7%), which suggests ChatGPT performs at or near the level of humans on the ASSH SAE. Despite the new image processing feature for ChatGPT, GPT-Hand was unable to use the image inputs to perform better on image-based questions. In fact, GPT-Hand only performed 5.4% better (P > 0.05) when provided clinical and radiographic image inputs on image-based questions. Additionally, there were no significant differences in the performance of GPT-4 versus GPT-Hand based on question difficulty or question topic. Even when provided with the facts required to answer a question correctly, GPT-Hand was not able to integrate this information to answer second- or third-order questions that require clinical reasoning. Overall, customized training increased performance on text-based questions, but ChatGPT still has significant shortcomings regarding image interpretation, which is essential for implementing this technology into clinical practice and education for hand surgery.

This study builds upon our previous work by introducing a customized GPT model (GPT-Hand) trained specifically with hand surgery resources [15]. This allows for a more focused and specialized evaluation compared to the broader, general-purpose GPT models used in our earlier study. Unlike the previous manuscript, which primarily assessed the performance of GPT-3.5 and GPT-4 on text-only questions, this submission evaluates GPT-Hand’s ability to handle both text and image-based questions from the 2022 ASSH SAE, providing a more comprehensive analysis of the model’s capabilities. The inclusion of image-based questions and the comparison of GPT-Hand’s performance with and without image inputs are significant additions, addressing a key limitation of the prior study. These enhancements demonstrate the customized model’s improved accuracy in text-based questions while also highlighting its ongoing limitations in image interpretation, a critical area not fully explored in the earlier research. Therefore, this manuscript offers a deeper, more nuanced understanding of the potential and limitations of customized AI models in hand surgery education, differentiating it clearly from our earlier publication.

For text applications, these models may soon be ready for real-world use as clinical and educational tools [2,6]. The performance on text-only questions following training with peer-reviewed knowledge indicates that, if developed using resources created by hand surgery experts, ChatGPT could serve as a useful tool. However, there were three text questions that GPT-4 answered correctly and the customized GPT answered incorrectly. Even when provided with peer-reviewed articles that contain the answer to a question, the model was unable to successfully process and apply that information. This may be due to conflicting evidence between the baseline information available to GPT-4 and that provided to GPT-Hand through training or by the intrinsic stochasticity intentionally built into ChatGPT [25]. Future work should continue to assess the consistency of the responses of trained models.

On image-based questions, providing the associated image did not result in a significant difference in performance. ChatGPT is not yet capable of effectively analyzing and applying information from the set of images included in the ASSH SAE. When providing MLLMs such as ChatGPT with training, it is necessary to consider this poor image interpretation capacity as a limitation for their utility in clinical practice and education. Images present in training materials are unlikely to be integrated effectively into the fund of training knowledge. Clinicians customizing MLLMs in the future might alternatively include text-based descriptions of images.

Neither question taxonomy (P = 0.317) nor subject category (P = 0.317) had a significant impact on the probability of a correct response for the customized GPT for all questions. The discrimination index, however, did predict customized GPT performance, whereby the questions that discriminated between the highest- and lowest-performing humans were less likely to be answered correctly. However, the increased performance on lower discrimination index questions raises concerns about how the correct answer was chosen by GPT-Hand.

Research on the application of ChatGPT in medicine has primarily focused on the analysis of its output [9,17,26], performance on standardized examinations [11-14,16], and the ability to process media [23]. ChatGPT has exhibited promising results for effectively answering commonly asked questions regarding distal radius fracture care, but authors have cautioned users due to the inconsistency in the accuracy of its answers [17]. Our results showed improved performance compared to a study by Han et al., which utilized ASSH SAEs from 2004 to 2013 [16]. Han et al. showed that ChatGPT-3.5 had a 39.2% correct response rate on text-based questions and a 28.7% correct response rate on image-based questions [16]. It is important to note that ChatGPT-3.5 did not have the ability to include image inputs, so the responses to image-based questions solely relied on the text-based inputs in the study by Han et al. Alternatively, Arango et al. found that GPT-4 scored 72.9% on the 2022 SAE, meeting the 50% correct answer rate required for orthopedic and plastic surgeons to receive CME and MOC credit [15]. This positive trend in performance may reflect advancements in ChatGPT or greater familiarity with current topics. The results of our study suggest training ChatGPT with additional relevant information appears to improve the overall performance for specific questions. Regarding a customized MLLM, previous work conducted by Fisher et al. found a model trained in the field of anesthesiology to perform with 90.8% accuracy [27], a finding consistent with ours. That study employed qualitative methods to evaluate the responses of the chatbot to 11 questions for accuracy and completeness [27]. The current study, however, is the first to our knowledge to quantitatively evaluate a customized GPT’s performance on hand surgery topics.

This study found GPT-Hand’s current image interpretation ability to be poor, answering 75.3% of image-based questions correctly. Posner et al. observed GPT-4 to answer 47.59% of image-based questions correctly in their study on effective medical image processing [23]. Shifai et al. evaluated GPT-4’s accuracy in the histopathological diagnosis of melanoma, reporting an overall accuracy of 36% [28]. Xu et al. assessed GPT-4’s ability to analyze multimodal ophthalmic images, recording a 30.6% correct response rate [29]. The comparatively greater accuracy we observed could be attributed to GPT-Hand’s ability to arrive at the answer using just the text from the image-based questions; however, it may also represent an improvement in the technology. Future work should continue to evaluate image interpretation in the presence and absence of accompanying text. ChatGPT is evolving to include non-text input interpretation, but existing technologies, such as convolutional neural networks, were constructed for the purpose of image processing and are currently superior for medical image interpretation purposes [30]. Although MLLMs have the potential to become powerful multimodal diagnostic tools, the use of alternative, image-focused AI technologies is currently recommended for clinical applications.

This study had multiple limitations. The investigation solely focused on the 2022 SAE and only utilized ChatGPT, limiting the generalizability of our findings to other MLLMs or examinations. We chose to only include the 2022 ASSH SAE as we had complete metrics of the human examinees that took the examination, while these data were not available for other examination years. Second, intra-model consistency was not addressed in this investigation. Finally, this study did not include an assessment of the model’s level of confidence in its output. Assessment of confidence would entail requesting the model to rate its degree of certainty following each answer, allowing for more precise utilization of its responses. Selection bias was minimized in this study through the inclusion of both text and image questions; however, video-based questions were excluded as ChatGPT is unable to interpret video inputs.

## Conclusions

ChatGPT’s performance can be improved and optimized for hand surgery applications through customization; however, clinicians should proceed with caution. Our findings suggest significant improvement in text-based applications with training ChatGPT using hand surgery resources. Despite this training, however, current image processing capabilities remain poor and require considerable improvements before having a role in education and clinical applications in hand surgery. This research also highlights the necessity of collaboration between AI system developers and hand surgeons to create effective educational and clinical MLLM tools.
